# Patulin Degradation by the Biocontrol Yeast *Sporobolomyces* sp. Is an Inducible Process

**DOI:** 10.3390/toxins9020061

**Published:** 2017-02-10

**Authors:** Giuseppe Ianiri, Cristina Pinedo, Alessandra Fratianni, Gianfranco Panfili, Raffaello Castoria

**Affiliations:** 1 Department of Agricultural, Environmental and Food Sciences, Università degli Studi del Molise, via Francesco de Sanctis, 86100 Campobasso, Italy; cristina.pinedo@uca.es (C.P.); fratianni@unimol.it (A.F.); panfili@unimol.it (G.P.); 2 Department of Organic Chemistry, Universidad de Cádiz, 11510 Puerto Real (Cádiz), Spain

**Keywords:** *Sporobolomyces* sp. IAM 13481, microbial patulin degradation, desoxypatulinic acid, ascladiol

## Abstract

Patulin is a mycotoxin produced by *Penicillium expansum* and a common contaminant of pome fruits and their derived products worldwide. It is considered to be mutagenic, genotoxic, immunotoxic, teratogenic and cytotoxic, and the development of strategies to reduce this contamination is an active field of research. We previously reported that *Sporobolomyces* sp. is able to degrade patulin and convert it into the breakdown products desoxypatulinic acid and ascladiol, both of which were found to be less toxic than patulin. The specific aim of this study was the evaluation of the triggering of the mechanisms involved in patulin resistance and degradation by *Sporobolomyces* sp. Cells pre-incubated in the presence of a low patulin concentration showed a higher resistance to patulin toxicity and a faster kinetics of degradation. Similarly, patulin degradation was faster when crude intracellular protein extracts of *Sporobolomyces* sp. were prepared from cells pre-treated with the mycotoxin, indicating the induction of the mechanisms involved in the resistance and degradation of the mycotoxin by *Sporobolomyces* sp. This study contributes to the understanding of the mechanisms of patulin resistance and degradation by *Sporobolomyces* sp., which is an essential prerequisite for developing an industrial approach aiming at the production of patulin-free products.

## 1. Introduction

Mycotoxins are secondary metabolites produced by fungi belonging to several genera, such as *Aspergillus, Penicillium*, and *Alternaria*. Besides their economical importance due to crops infection and product losses, their toxic secondary metabolites represent a health risk to humans and animals. The maximum content of some mycotoxins in harvested commodities and derived products has been established by national and international organizations, and the reduction of mycotoxin contamination is an important research focus.

Patulin (PAT) is a toxic fungal metabolite produced mainly by species of *Penicillium* and *Aspergillus*. *P. expansum*, the causative agent of the blue mould disease of stored apples, is the main PAT producer and its infections result in PAT contamination of apples, pears, and their derived products [1]. The carcinogenic risk of PAT is classified in group 3 by the International Agency for Research on Cancer [2], and this has led to the establishment of a maximum tolerable daily intake for PAT of 0.4 mg/kg body weight/day [3]. Moreover, although there are legislative regulations in Europe and USA that set the highest tolerable levels of PAT in fruit-based products and juices at 50 μg/kg, and for baby food at 10 μg/kg (EC Regulation 1881/2006), recent surveys in Europe and USA revealed that PAT contamination is still a common issue [4,5,6].

The mechanisms of PAT toxicity and its effects on living cells have been long investigated [1,7]. Patulin was found to have antibacterial and antifungal activities [7]. In mammals, the primary target organs of PAT toxicity are the gastrointestinal tract, kidney, liver and the immune system. Continued exposure to high concentrations of PAT may include mutagenicity, genotoxicity, embryotoxicity, immunotoxicity and neurotoxicity [1,7,8].

The ecological role of PAT has not been fully elucidated, and it is likely to help in competition against other microbes that PAT-producing fungi encounter in their niche. Mutation in the gene encoding enzymes involved in PAT biosynthesis [7,9] has allowed to investigate the role of PAT in the pathogenicity of *P. expansum*, which is still controversial and it seems to be dependent on factors of the host fruits [10,11,12,13].

Limiting *P. expansum* infections in postharvest settings is crucial to prevent PAT accumulation. The use of chemical fungicides is still the most effective approach. However, ethical, technical and health issues, foster an increasing demand for alternative methods to reduce the use of chemicals, and the use of biocontrol agents and/or controlled atmosphere are very promising strategies [6]. Moreover, their combination with tools aiming at the detoxification of PAT has the potential to further reduce PAT contamination, thus providing safer fruit juices; an important focus because their contamination poses a major risk for children, who consume great quantities of fruit juices.

The influence of biocontrol agents (BCAs) that are effective against *P. expansum* on PAT accumulation is an emerging and attractive field of study, and major findings have been elegantly reviewed [14]. The ability of PAT degradation seems to be a widespread feature of the *Pucciniomycotina* red yeasts. We found that *Sporobolomyces* sp. IAM 13481 was able to convert PAT to DPA and ascladiols, with (*E*) ascladiol being a transient metabolite, and DPA and (*Z*) ascladiol the two final breakdown products [15]. In the red yeast BCAs *Rhodotorula kratochvilovae* and *R. paludigenum*, DPA was found as the major PAT degradation products [16,17,18], although at least in the case of *R. kratochvilovae*, the isomers (*E*) and (*Z*) of ascladiol were found as transient products (our unpublished data). Using *Sporobolomyces* sp. IAM 13481 as a model, we explored the molecular mechanisms involved in PAT degradation by *Pucciniomycotina* yeasts through a random insertional mutagenesis approach based on *Agrobacterium tumefaciens*-mediated transformation (AMT) [15]) as well as a transcriptomic approach based on RNAseq analysis of yeast cells grown in the presence and absence of this mycotoxin [19].

Our data indicated that PAT toxicity is exerted through the generation of ROS (reactive oxygen species), which leads to cellular damage but also function as signalling molecules that activate cellular components required for oxidative stress resistance and PAT degradation. Based on these findings, in the present work, we investigated whether the biochemical mechanisms that lead to PAT resistance and degradation in *Sporobolomyces* sp. IAM13481 can be induced by exposition to low doses of this mycotoxin.

## 2. Results

### 2.1. Pre-Treatment of Sporobolomyces sp. IAM 13481 Cells with Patulin Induces Mechanisms Involved in Its Resistance and Degradation

**Figure 1:**
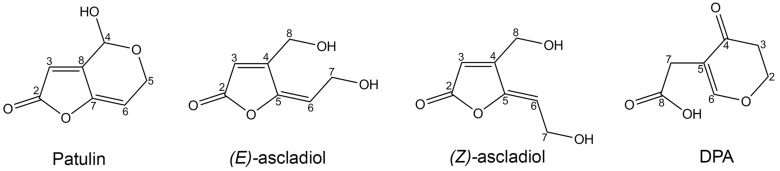
Chemical structures of patulin and its breakdown products (*E*) and (*Z*) ascladiol, and desoxypatulinic acid (DPA).

*Sporobolomyces* sp. IAM 13481 is able to degrade in vitro the mycotoxin PAT under aerobic conditions. The products of PAT degradation have been identified as (*E*)-ascladiol, (*Z*)-ascladiol and desoxypatulinic acid (DPA); (*E*)-ascladiol is a transient or less stable product, while DPA and (*Z*)-ascladiol are the final metabolites of PAT degradation [15] (Figure 1).

In the present work, the induction of the mechanisms that underlie PAT resistance and degradation by *Sporobolomyces* sp. IAM 13481 was assessed by incubating pretreated cells (PC, cells preincubated in the presence of 15 μg/mL of PAT) and non-preincubated cells (NPC, cells incubated in the absence of PAT) in the presence of two different concentrations of PAT, 50 and 100 μg/mL.

**Figure 2:**
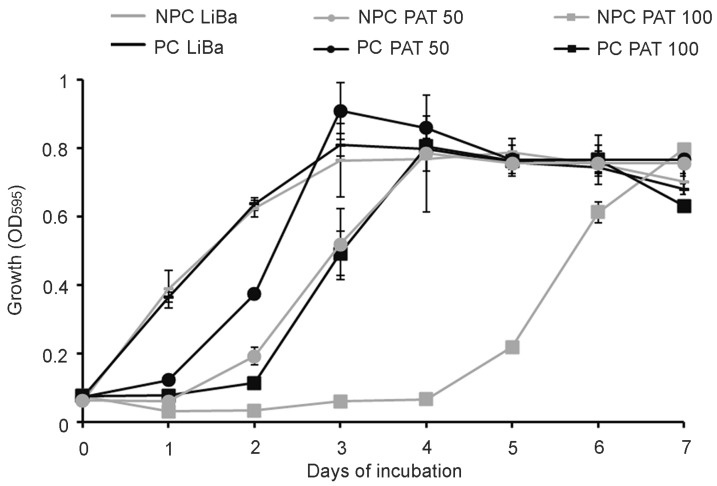
Growth curves of *Sporobolomyces* sp. IAM 13481 cells that were preincubated (PC, black lines) and non-preincubated (NPC, grey lines) with PAT in the presence (50 and 100 μg/mL) or in the absence (i.e., LiBa alone) of the mycotoxin. Values are expressed as OD_595_ and represent the means ± standard deviation (*n* = 9).

In Figure 2 the growth curves of NPC and PC of *Sporobolomyces* sp. IAM 13481 in the absence and in the presence of 50 and 100 μg/mL of PAT are reported. While in LiBa without PAT the growth kinetics of NPC and PC followed a similar trend, there were clear differences between their growth in the presence of the mycotoxin. In particular, with 50 μg/mL of PAT, PC suffered from the initial toxicity of PAT only partially and reached the highest OD_595_ value after 3 days of incubation, whereas the growth of NPC was characterized by a 1-day lag phase followed by exponential growth until the fourth day of incubation. In the presence of 100 μg/mL of PAT, there were more marked differences between the growth of PC and NPC, with PC showing a 1-day lag phase followed by active growth until the fourth day of incubation, a trend that reflected that of NPC grown with PAT 50 μg/mL, and NPC characterized by a 4-day lag phase followed by exponential growth until the seventh day of incubation.

**Figure 3:**
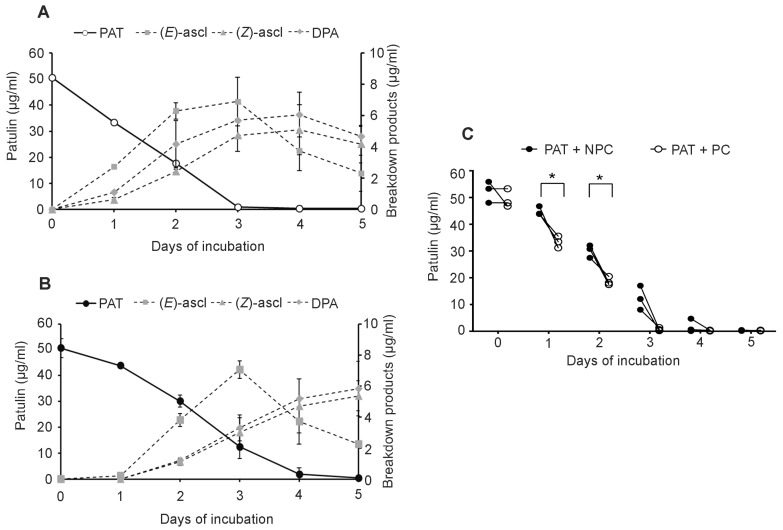
Time course of PAT decrease (full line) and onset of breakdown products (dotted lines) of *Sporobolomyces* PC (**A**) and NPC (**B**) grown in the presence of 50 μg/mL of mycotoxin. PAT is patulin, (*E*)-ascl and (*Z*)-ascl are (*E*)-ascladiol and (*Z*)-ascladiol, respectively. Values are expressed as μg/mL and represent the means ± standard deviation (*n* = 9, three measurements in three experiments); (**C**) Fate of 50 μg/mL of PAT in the presence of *Sporobolomyces* PC and NPC. Asterisk indicates significant differences (*p* < 0.05) when analyzed by Student’s *t* test.

In accordance with our previous report [15], the kinetics of PAT degradation reflected the growth curves both for PC and NPC. As reported in Figure 3, with 50 μg/mL of PAT, after 24 h of incubation there was a significant difference between the PAT recovery of PC and NPC, with values of 33.40 ± 2.16 μg/mL and 43.91 ± 0.11 μg/mL, respectively (Figure 3A–C). At the same time point, PC produced 2.73 ± 0.14 μg/mL of (*E*)-ascladiol, 0.62 ± 0.15 μg/mL of (*Z*)-ascladiol and 1.09 ± 0.04 μg/mL of DPA (Figure 3A), while only traces of (*E*)-ascladiol were detected for NPC (Figure 3B). Also, at 48 h of incubation there was a significant difference in PAT reduction by *Sporobolomyces* PC and NPC (Figure 3C). PC led to a complete degradation of 50 μg/mL of PAT after three days of incubation, in correspondence of which they produced 6.90 ± 1.56 μg/mL of (*E*)-ascladiol, 4.47 ± 1.04 μg/mL of (*Z*)-ascladiol and 5.70 ± 0.04 μg/mL of DPA. In the same culture, at the fourth day of incubation, the (*E*)-ascladiol decreased to 3.75 ± 1.27 μg/mL, whereas the (*Z*)-ascladiol and DPA increased respectively to 5.09 ± 1.61 μg/mL and 6.06 ± 1.43 μg/mL, the highest values achieved; at the last time points, the amount of the breakdown products slightly decreased (Figure 3A) probably due to their instability or other unknown enzymatic activities. In NPC of *Sporobolomyces*, after four days of incubation, PAT was almost totally degraded (PAT was only 1.81 ± 2.52 μg/mL), and this paralleled the production of 5.36 ± 3.44 μg/mL of (*E*)-ascladiol, 4.70 ± 1.75 μg/mL of (*Z*)-ascladiol and 5.15 ± 1.30 μg/mL of DPA; at the fifth day, the (*E*)-ascladiol decreased, while (*Z*)-ascladiol and DPA slightly increased (Figure 3B).

**Figure 4:**
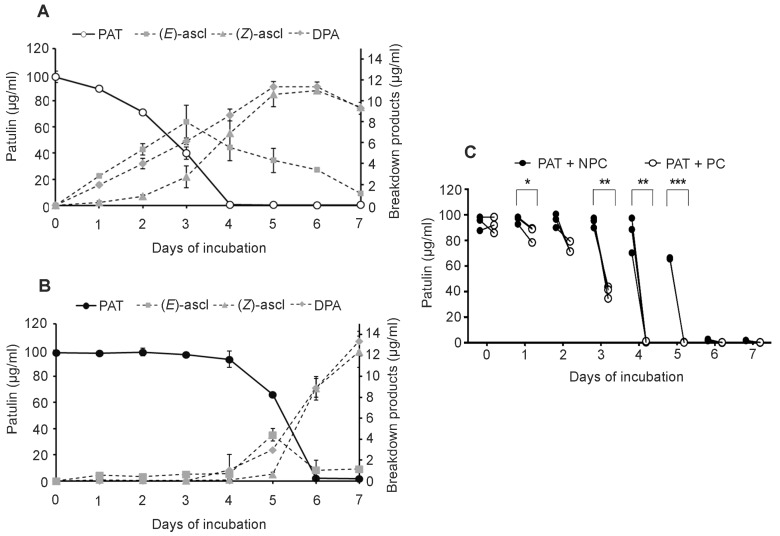
Time course of PAT decrease (full line) and onset of breakdown products (dotted lines) of *Sporobolomyces* PC (**A**) and NPC (**B**) grown in the presence of 100 μg/mL of PAT. PAT is patulin, (*E*)-ascl and (*Z*)-ascl are (*E*)-ascladiol and (*Z*)-ascladiol, respectively. Values are expressed as μg/mL and represent the means ± standard deviation (*n* = 9, three measurements in three experiments); (**C**) Fate of 100 μg/mL of PAT in the presence of *Sporobolomyces* PC and NPC. Symbols indicate significant differences for *p* < 0.05 (*), *p* < 0.01 (**), and *p* < 0.001 (***) when analyzed by Student’s *t* test.

With 100 μg/mL of PAT (Figure 4), PC of *Sporobolomyces* started to degrade the mycotoxin since the first day of incubation (PAT recovery of 89.18 ± 0.38 μg/mL), and this paralleled the production of about 2 μg/mL of (*E*)-ascladiol and DPA, and traces of (*Z*)-ascladiol (Figure 4A). At the third day of incubation, more than 50% of PAT was degraded, and the highest production of (*E*)-ascladiol (8.00 ± 0.35 μg/mL) was also achieved, which, as expected, decreased to 1.54 ± 0.41 μg/mL on the last day of incubation. PAT was completely degraded after four days of incubation, and, at the same time point, the amount of the final breakdown products (*Z*)-ascladiol and DPA were 6.91 ± 1.75 μg/mL and 8.64 ± 0.55 μg/mL, respectively. Even though no more PAT was present in the medium, the amounts of these two degradation products still increased to reach the highest values at the sixth day of incubation, 11.00 ± 1.66 μg/mL and 11.34 ± 0.44 μg/mL for (*Z*)-ascladiol and DPA, respectively (Figure 4A). For NPC instead (Figure 4B), a considerable PAT reduction of about 40% was observed only at the fifth day of incubation, and at the same time point, (*E*)-ascladiol reached its production peak (4.42 ± 0.62 μg/mL) and decreased in the following days, while the amounts of (*Z*)-ascladiol and DPA were still low (traces for (*Z*)-ascladiol and 2.99 ± 0.11 μg/mL for DPA). PAT was completely degraded at six days of incubation, and at the seventh day there was the highest production of (*Z*)-ascladiol and DPA (12.36 ± 1.49 μg/mL and 13.36 ± 0.96 μg/mL, respectively) (Figure 4B). As shown in Figure 4C, after 1, 3, 4 and 5 days of incubation, there were highly significant differences between the degradation of 100 μg/mL of PAT by PC and NPC. With 200 μg/mL of PAT, there were even more remarkable differences between PC and NPC of *Sporobolomyces*, and when there was a lower initial concentration of cells (i.e., 1 × 10^5^ CFU/mL), only PC were able to grow and degrade PAT, whereas NPC did not (data not shown).

### 2.2. Intracellular Extracts from Sporobolomyces sp. Cells Preincubated with Patulin Degrade the Mycotoxin More Rapidly Than Extracts from Non-Preincubated Cells

Intracellular extracts from PC (EPC, induced extracts) were obtained from *Sporobolomyces* sp. cells in their active phase of PAT degradation, which was monitored by TLC analysis as described in Section 5.5; intracellular extracts from non-preincubated cells (ENPC) were obtained from *Sporobolomyces* sp. cells grown in LiBa medium without PAT when the growth reached the same OD_595_ as the culture with PAT. Using this rationale, cells for intracellular extracts were expected to be at the same metabolic stage, with the only difference being the presence or the absence of the mycotoxin.

**Figure 5:**
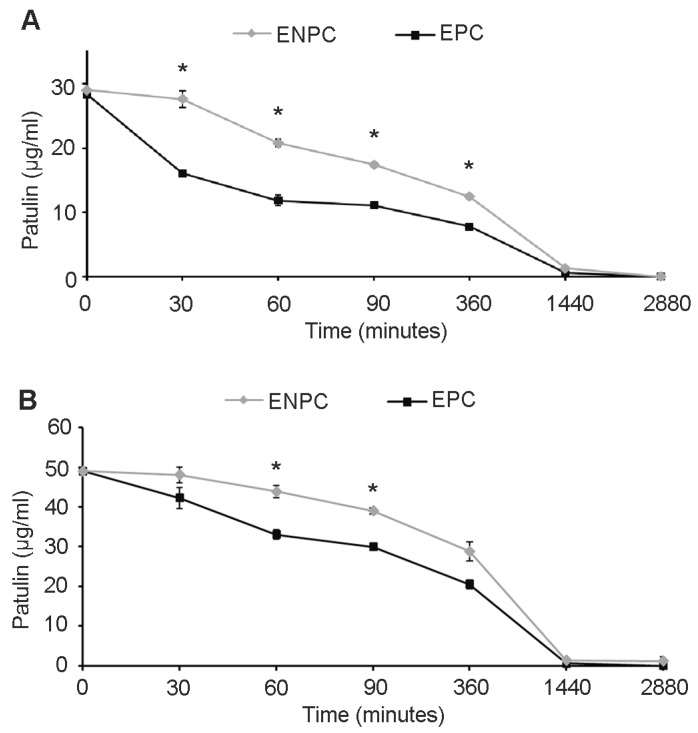
Fate of 30 μg/mL (**A**) and 50 μg/mL (**B**) of PAT in the presence of induced intracellular extracts (EPC, black lines) and non-induced intracellular extracts (ENPC, grey lines) of *Sporobolomyces*. Values are expressed as μg/mL and represent the means ± standard deviation (*n* = 9, three measurements in three experiments). Symbols indicate significant differences for *p* < 0.05 (*) when analyzed by Student’s *t* test.

In Figure 5A,B, the fate of 30 and 50 μg/mL of PAT in the presence of EPC and ENPC of *Sporobolomyces* sp, respectively, is reported. With 30 μg/mL of PAT, even after 30 min of incubation, there was a significant difference between EPC and ENPC, with a PAT recovery of 16.09 ± 0.13 μg/mL and 27.68 ± 1.30 μg/mL, respectively; from 60 min to 360 min (6 h) of incubation; the differences in PAT recovery between EPC and ENPC were still significant, and in both cases only traces of mycotoxin were found after 1440 min (24 h) of incubation (Figure 5A). When 50 μg/mL of mycotoxin was used, significant differences between the PAT recovery in the presence of EPC and ENPC were found after 60 and 90 min of incubation, and since then they were progressively lower until only traces of mycotoxin were found after 1440 min of incubation (Figure 5B). At both concentrations used (30 μg/mL and 50 μg/mL), no PAT decrease was observed when incubated in the presence of boiled ENPC and EPC.

## 3. Discussion

PAT is a worldwide mycotoxin that represents an economic challenge to pome fruit food industries and a health hazard to consumers. The reduction of PAT contamination through the use of beneficial microbes has been known for long time and it is still an active field of research [14].

*Pucciniomycotina* red yeasts have emerged amongst the microbes that exhibit an elevated tolerance to PAT toxicity and have a constitutive arsenal to breakdown the PAT molecule. Previous research showed that the red yeast *Sporobolomyces* sp. IAM 13481 used in the present study is able to degrade PAT in vitro by forming desoxypatulinic acid (DPA) and (*Z*)-ascladiol as the final products of degradation, whereas (*E*)-ascladiol is only formed as a transient product [15]. The red yeasts *Rhodotorula kratochvilovae* LS11 and *R. paludigenum* (originally classified as *Rhodosporidium* but renamed following recent taxonomy analysis [20]) are also able to degrade the mycotoxin PAT, but only DPA was found as a final product of degradation [17,18]; at least for *R. kratochvilovae* LS11, both ascladiol isomers were detected as transient products (our unpublished data). We also found that several other red yeast species degrade PAT in a similar manner to *Sporobolomyces* or *Rhodotorula* (our unpublished data).

Therefore, it seems likely that *Pucciniomycotina* red yeasts degrade PAT through two different pathways, and their regulation differs between the species under examination. The hypothesis of the two distinct pathways is supported by the chemical structures of DPA and ascladiol, as they derive from the breakage of bonds that are located in separate ends of the PAT molecule (Figure 1). Interestingly, the production of DPA has been described only in red yeasts, whereas ascadiol was found following PAT degradation both by yeasts (i.e., *Saccharomyces cerevisiae*) and bacteria (*Gluconobacter oxydans* and *Lactobacillus plantarum* [21,22,23]). This suggests that while the ascladiol-forming degradation pathway is likely to be conserved across fungi and bacteria, the DPA-forming pathway has independently evolved in the red yeast group within the *Pucciniomycotina*. The importance of these mechanisms of degradation is supported by the low toxicity of the PAT breakdown products: DPA is less toxic to different microorganisms, to human lymphocytes and hepatocytes, as well as to *Arabidopsis thaliana* [17,18,24,25]; ascladiol was originally reported as retaining a quarter of the toxicity displayed by PAT [26], and a more recent investigation demonstrated that it is not toxic to the porcine intestinal tissue [27].

Noteworthy, the sum of the breakdown products was found to be less than the initial concentration of PAT. Although there is evidence of adsorption of PAT on the yeast cell wall [28], this phenomenon does not occur in *Sporobolomyces* sp. [15], *R. paludigenum* [17] and *R. kratochvilovae* [18], indicating that beside the ascladiol- and DPA- forming degradation pathways, in red yeasts part of the mycotoxin is probably transformed in other compounds that can enter other metabolic pathways for energy or other cellular functions, or might be internalized in the cell and/or in organelles.

In the present work, we further examined the response of *Sporobolomyces* to PAT and demonstrated that the resistance to and/or degradation of the mycotoxin by *Sporobolomyces* sp. IAM 13481 are inducible processes. In particular, *Sporobolomyces* cells that were pretreated (PC) with a low concentration of PAT showed a faster growth (Figure 2) and a faster kinetics of PAT degradation compared to *Sporobolomyces* cells that were not preincubated with PAT (NPC) (Figure 3 and Figure 4), with differences being more evident when high concentrations of PAT (100 or 200 μg/mL versus 50 μg/mL) were used after the preincubation. Counts of viable *Sporobolomyces* cells after 1 day of incubation revealed that with 50 μg/mL of PAT, the growth of PC and NPC increased in a similar manner (5.3 × 10^7^ CFU/mL and 4.0 × 10^7^ CFU/mL, respectively) compared to the initial cellular concentration (i.e., 1 × 10^7^ CFU/mL). Conversely, in the presence of 100 μg/mL and 200 μg/mL of PAT, the growth of NPC dropped respectively to 1.7 × 10^5^ and 4.3 × 10^3^ CFU/mL, with that of PC being instead 1.2 × 10^7^ and 6.4 × 10^5^ CFU/mL, respectively. Taken together, these data indicate that *Sporobolomyces* PC have the highest tolerance to the initial stresses caused by PAT toxicity and this resulted in a faster growth and degradation of the mycotoxin. This is also demonstrated by the similar growth (Figure 2) and kinetics of PAT degradation of *Sporobolomyces* NPC incubated with 50 μg/mL of mycotoxin (Figure 3B) and *Sporobolomyces* PC incubated with 100 μg/mL of mycotoxin (Figure 4A). Similar results were also obtained for *R. kratochvilovae* LS11 (our unpublished data) and *S. cerevisiae* [29]. Based on our recent RNAseq study performed with *Sporobolomyces* sp. IAM 13481 incubated in the presence or absence of 5 μg/mL and 50 μg/mL of PAT [19], it is likely that the pre-incubation step with the mycotoxin induces the earlier activation of (i) genes encoding proteins involved in response to oxidative and other stresses related to PAT cytotoxicity; (ii) antioxidants systems (glutathione and thioredoxin) essential for restoring the cellular redox homeostasis; (iii) export and detoxification proteins that are predicted to be involved in PAT efflux and PAT degradation. Therefore, *Sporobolomyces* PC cells are already in an active/alert stage that enables them to better counteract the stronger toxicity impact due to the use of higher PAT concentrations. Other data that corroborate this interpretation of the obtained results come from our screening of T-DNA insertional mutants of *Sporobolomyces* sp. IAM 13481; we found that the inactivation of genes involved in the resistance to oxidative, genotoxic and other stresses caused by PAT resulted in an increased sensitivity of the mutants to the mycotoxin as compared to the wild type strain [15].

A caveat of using intact cells of *Sporobolomyces* sp. IAM 13481 consisted in the difficulty in evaluating the induction of mechanisms involved in the direct degradation of the PAT, which might be hidden by the multiplicity of resistance mechanisms activated in response to the mycotoxin. Therefore, with the rationale to focus only on the degradation mechanisms and excluding those of resistance, the fate of PAT was also tested in the presence of intracellular proteins extracted from *Sporobolomyces* sp. IAM 13481 cells grown in the presence (EPC) and in the absence of PAT (ENPC). Results presented in Figure 5A,B showed that EPC degrades PAT more promptly as compared to ENPC, although in all cases there were no differences in the length of the degradation period as only traces of PAT were detected after 24 h of incubation (1440 min). Corroborating data obtained for *R. kratochvilovae* LS11 (our unpublished data), *R. paludigenum* [17] and *S. cerevisiae* [29], our results confirm that PAT degradation is an enzyme-mediated mechanism and that the synthesis of the relevant enzyme(s) is likely induced by PAT treatment, further suggesting that PAT metabolization is itself a mechanism of resistance that contributes to overcoming PAT toxicity together with the activation of defense mechanisms described above. Several genes encoding for enzymes specifically involved in PAT degradation have been predicted [19], and they include a glucose–methanol–choline (GMC) oxidoreductase, a protein subunit of aromatic ring-opening dioxygenase, the vacuolar proteins Env9 and Ycf1, and several short and medium chain dehydrogenases. Their target mutagenesis is in progress to elucidate their role in the hydrolysis of the mycotoxin, and to allow the identification of the cellular and molecular components that control their expression. Last but not least, the evaluation of the biocontrol activity of these mutants against *P. expansum* will help to understand whether PAT degradation is also an additional mechanism of action used by the biocontrol agents to counteract postharvest pathogens, since some recent reports point to PAT as a factor involved in the virulence/aggressiveness of *P. expansum*, at least on some cultivars of apples [13].

## 4. Conclusions

In conclusion, in the present study, we used the model organism *Sporobolomyces* sp. IAM 13481 to evaluate the induction of mechanisms of PAT resistance and degradation; the induction step was performed by pretreating *Sporobolomyces* cells with a low concentration of PAT. Our results suggest that intact yeast cells were appropriate for the evaluation of the mechanisms of PAT resistance and degradation together, while the use of intracellular extracts allowed to evaluate more specifically the mechanisms of PAT degradation. We found that the mechanisms that control both PAT resistance and degradation can be induced by pretreatment with the mycotoxin, and that both processes are related to each other and overall, they contribute both to reduce PAT concentration and mitigate its toxicity. These results contribute to the achievements of long-term goals, namely the production of the enzymes responsible for PAT detoxification that could be the base for detoxification processes of products and juices derived from pome fruits.

## 5. Materials and Methods

### 5.1. Strain Used in This Study

The red yeast *Sporobolomyces* sp. strain IAM 13481 was used in this study. This strain was routinely cultivated on yeast peptone dextrose (YPD: Yeast extract 10 g/L, Peptone 20 g/L, Glucose 20 g/L, Agar 20 g/L).

### 5.2. Patulin

Commercial standards of PAT were purchased from Sigma-Aldrich (Milan, Italy) and from A.G. Scientific, Inc. (San Diego, CA, USA). PAT was dissolved in ethyl acetate and stored at −20 °C. For experiments, the appropriate aliquots of PAT were withdrawn and the ethyl acetate evaporated under nitrogen stream. The dry PAT was subsequently resuspended at the desired concentrations in working solutions that were LiBa medium (LiBa, 10.0 g of d-glucose, 2.0 g of l-asparagine, 1.0 g of KH_2_PO_4_, 0.5 g of MgSO_4_·7H_2_O, 0.01 mg of FeSO_4_·7H_2_O, 8.7 mg of ZnSO_4_·7H_2_O, 3.0 mg of MnSO_4_·H_2_O, 0.1 mg of Biotin, and 0.1 mg of Thiamine, per Liter) [30] for assays with intact cells, or in buffer potassium phosphate 0.1 M pH 6 [31] for assays with intracellular extracts. The PAT working solutions were filter-sterilized (0.2 μm) prior to use.

### 5.3. Patulin Degradation Assays with PC and NPC of Sporobolomyces sp. IAM 13481

Analysis of PAT persistence in the presence of PC and NPC of *Sporobolomyces* sp. was performed as previously reported [15] with minor modifications. Cells of *Sporobolomyces* sp. were grown overnight in LiBa medium in the absence and in the presence of 15 μg/mL of PAT. Cultures were centrifuged for 5 min at 4000 rpm, the cells were resuspended in LiBa, and their concentration was adjusted to 1 × 10^7^ CFU/mL. Three millilitres of this suspension was incubated at 24 °C in sterile 25 mL-flasks in the presence of 50 and 100 μg/mL of PAT. Yeasts growth was monitored on a daily basis by reading OD at 595 nm in a microplate Reader (Bio-Rad Laboratories, Hercules, CA, USA) [32], and at the same time points, the time-course of PAT degradation was assessed by means of HPLC analysis in the same samples as described in Section 5.5. Data from the experiments were pooled since they were similar in the three repetitions.

### 5.4. Patulin Degradation Assays Using Intracellular Protein Extracts Obtained from PC and NPC of Sporobolomyces sp. IAM 13481

In order to obtain induced intracellular extracts of *Sporobolomyces* sp. IAM 13481, an overnight culture of yeast cells was diluted to 1 × 10^7^ CFU/mL, inoculated in LiBa medium with 15 μg/mL of PAT and incubated on a rotary shaker at 24 °C. The yeast growth was monitored by reading the OD at 595 nm, and the degradation of PAT and the onset of breakdown products were monitored by TLC analysis as previously described [15,16]. When the intensity decrease of the PAT spot paralleled the onset of the degradation products, achieved at OD_595_ of ~0.25, intracellular proteins were extracted as described below. Non-pretreated intracellular samples were extracted from *Sporobolomyces* sp. cells grown in LiBa (same initial concentration as in PC) without PAT until the OD_595_ value reached the same value as the culture grown in the presence of the mycotoxin.

Cells were collected by centrifugation for 10 min at 4000 rpm and then lyophilized. Five hundred milligrams of lyophilized pellets was resuspended with lysis buffer [buffer potassium phosphate 50 mM pH 6 + phenylmethylsulfonyl fluoride (PMSF) 1 mM], 300 mg of acid-washed glass beads was added and the samples vortexed for 1 min. Samples were kept in liquid nitrogen for 1 additional min, then thawed and subsequently centrifuged at 10,000 rpm for 5 min at 4 °C. Supernatants were kept on ice. This lysis step was repeated five times, at the end of which all the supernatants of the same sample were pooled. In order to eliminate residual pellet, the supernatants were filtered with 0.45 μm and 0.25 μm filters (Barloworld Scientific, Stone, Staffordshire, UK). Sodium azide (0.02% *w*/*v*) was added and the samples were dialyzed (dialysis tubes with cutoff 12,000 Da) in water (pH 6) for 24 h with three changes. Samples were lyophilized, suspended in buffer phosphate 50 mM pH 6 (without PMSF) and the intracellular proteins concentration was quantified using the Bradford assay (Bio-Rad) according to the manufacturer’s instructions, with the BSA used as reference.

PAT degradation assays were carried out in 50 mL falcon tubes each containing 3 mL of incubation mixture. Tubes were kept on a rotary shaker at 160 rpm and 24 °C. Amounts of 30 and 50 μg/mL of PAT were added to phosphate buffer 50 mM pH 6 containing 0.4 mg/mL of intracellular extracts. Controls were PAT in buffer phosphate, and PAT incubated in the presence of boiled intracellular extracts. PAT fate was assessed by HPLC analysis according to the same conditions as those described above after 30 min, 60 min, 90 min, 6 h (360 min), 24 h (1440 min) and 48 h (2880 min). At each time point, 100 μL of each suspension was withdrawn, extracted twice with ethyl acetate, dried and resuspended with buffer phosphate 50 mM pH 6 for HPLC analysis.

### 5.5. TLC and HPLC Analyses

TLC qualitative analysis and HPLC quantitative analyses were performed as previously reported [15,16,18].

For TLC analysis, samples were pooled, centrifuged at 14,000 rpm for 5 min for pelleting the cells, and the supernatant was extracted twice with ethyl acetate adjusted to pH 2. Extracted samples were dried under a nitrogen stream, resuspended in 10 μL of ethyl acetate and loaded on aluminium-backed silica gel 60 F254 plates (EMD Chemicals, Gibbstown, NJ, USA). Chromatography was performed at room temperature in glass tanks by using toluene/ethyl acetate/formic acid 5:4:1 (*v*/*v*/*v*) as the solvent system. After development, plates were dried, observed and photographed under UV light (λ = 254 nm).

Quantitative analysis of PAT and breakdown products formed by *Sporobolomyces* sp. IAM13481 was performed by HPLC based on MacDonald et al. [33] with slight modifications. Three samples per time point were centrifuged and filter-sterilized to remove cells, then injected for analysis. The HPLC apparatus was a Dionex (Sunnyvale, CA, USA) analytical system consisting of a P680 solvent delivery system and a 20 μL injector loop (Rheodyne, Cotati). The UVD170 detector (Dionex, Sunnyvale, CA, USA) set at 276 nm was connected to a data integration system (Dionex Chromeleon Version 6.6). Data from the experiments were pooled, since they were similar in the three repetitions, and expressed as μg/mL of patulin, desoxypatulinic acid and ascladiols ± standard deviation (*n* = 9).

### 5.6. Statistical Analysis

Patulin persistance in the biodegradation assays carried out using intact *Sporobolomyces* PC and NPC and their intracellular extracts (EPC and ENPC) was analyzed by Student’s *t* test using the software GraphPad Prism 7.00 for Mac (La Jolla, CA, USA), and differences were considered statistically significant when *p*-value was lower than 0.05.
